# Moving forward: scaling-up the integration of an HIV and hypertension program in Akwa Ibom State, Nigeria

**DOI:** 10.1186/s41256-024-00379-6

**Published:** 2024-09-14

**Authors:** Shivani Mishra, Angela A. Aifah, Daniel Henry, Nina Uzoigwe, Ememobong Bassey Udoh, Esther Idang, Jahnavi Munagala, Deborah Onakomaiya, Nafesa Kanneh, Anyiekere Ekanem, Eno Angela Attah, Gbenga Ogedegbe, Dike Ojji

**Affiliations:** 1 Institute for Excellence in Health Equity, NYU Grossman School of Medicine, New York, NY USA; 2 Section for Global Health, Department of Population Health, NYU Grossman School of Medicine, New York, NY USA; 3 Cardiovascular Research Unit, University of Abuja and University of Abuja Teaching Hospital, Gwagwalada, Abuja, Nigeria; 4 University of Uyo Teaching Hospital, Uyo, Akwa Ibom State Nigeria; 5 Akwa Ibom State Primary Healthcare Development Agency, Uyo, Akwa Ibom State Nigeria; 6 NYU Grossman School of Medicine, New York, NY, USA; 7 Vilcek Institute of Graduate Biomedical Sciences, NYU Grossman School of Medicine, New York, NY, USA; 8 Department of Internal Medicine, Faculty of Clinical Sciences, University of Abuja, Gwagwalada, Abuja, Nigeria

**Keywords:** HIV, Policy, Non-communicable diseases, Primary healthcare, Capacity building

## Abstract

As people living with HIV experience increased life expectancy, there is a growing concern about the burden of comorbid non-communicable diseases, particularly hypertension. This brief describes the current policy landscape on the management of HIV and hypertension in Akwa Ibom State, Nigeria, stakeholder engagement meetings, and the resulting five policy recommendations rooted in an ongoing research study designed to integrate hypertension management into HIV care across primary health centers in the State. In order to identify the current gaps in integrated care, discussion sessions with three stakeholder groups (i.e., healthcare providers, patient advocacy groups, and policy makers) were held separately in November 2022. The discussions were purposed to brainstorm policy-level solutions for integrating hypertension into HIV treatment. After all the sessions were concluded, there were five recommendations provided by the stakeholders for integrating HIV and hypertension care in the Akwa Ibom State. Stakeholders unanimously agreed with the need to integrate hypertension care for HIV impacted communities in the State. Specifically, stakeholders recommended to: (1) engage retired community health nurses as mentors; (2) actively link communities to integrated care in clinics; (3) integrate hypertension management with HIV education; (4) expand health insurance accessibility; and (5) formally integrate hypertension management into primary healthcare centers in Akwa Ibom State.

## Background

There are over 39 million people living with HIV (PLWH), and while the increased access to antiretroviral therapy (ART) has significantly reduced the mortality rate by nearly half from 2 million to 1 million deaths between the period of 2005 to 2016, there has been a significant increase in cardiovascular-related mortality [1]. Recent reports show that the global prevalence of hypertension, the leading risk factor for cardiovascular mortality among PLWH, was 23–24% [2]. While the African region has the lowest prevalence of both diagnosed and controlled hypertension, recent data shows increasing rates of cardiovascular diseases in PLWH [3]. Nigeria, the most populated country in Africa, has one of the highest rates of hypertension of PLWH [4]. Within Akwa Ibom State in Nigeria, a recent cross sectional study found the prevalence of hypertension among PLWH to be 24.9%, with only 24.4% of those having controlled hypertension [5]. Additionally, HIV treatment services are typically solitary programs throughout Africa. As a result, patients are burdened when accessing additional care for non-communicable disease (NCD) comorbidities, including transportation costs and medical expenses, contributing to higher morbidity and mortality among PLWH [6].

There is thus an urgent need to leverage available HIV care systems and resources to address the rising burden of NCDs in PLWH. Our research study, *Managing Hypertension Among People Living with HIV: an Integrated Model (MAP-IT)*, is designed to address this important problem. Its goal is to evaluate the adoption and sustainability of an integrated HIV and hypertension program among PLWH who receive care in primary health centers (PHCs) in Akwa Ibom State [7]—a state with one of the highest burdens in Nigeria. Specifically, MAP-IT provides hypertension care for PLWH through the existing HIV care services in PHCs across Akwa Ibom State [7]. As part of the task shifting strategy for hypertension, trained community health nurses implement the components of the program, which include identifying, counseling, treating, and referring PLWH with uncontrolled hypertension. The community health nurses are then mentored by trained and retired community health nurses who serve as practice facilitators [7]. 

Through MAP-IT, we identified key stakeholders who were part of the study’s steering committee (policymakers, patient advocacy groups, and healthcare workers) and solicited their feedback following pilot results. In November 2022, we organized three discussion sessions, each with a specific group of stakeholders. The first session was hosted for healthcare workers, the second for patient advocacy groups, and the third for policymakers. Discussions were purposed to brainstorm policy-level solutions to HIV-hypertension integration challenges.

Based on these three stakeholder discussions, this policy brief provides an overview of the current Nigerian policies that MAP-IT study leverages, the stakeholder-identified policy gaps in integrating HIV and hypertension care in Akwa Ibom State, and the corresponding policy-level solutions.

## Overview of current Nigerian policies the MAP-IT study leverages

MAP-IT focuses on two strategies (1) task-shifting of hypertension treatment duties from physicians to nurses, and (2) implementation of the program in PHCs using practice facilitation. As such, the three Nigerian policies relevant to the MAP-IT study are: the Task Shifting and Sharing policy, the Nigerian Hypertension Treatment Protocol, and the State Health Insurance Policy.

Nigeria has an established task-shifting and sharing policy (the “Task Shifting and Sharing Policy for Essential Health Care Services in Nigeria”) that provides access to vital health services through the efficient use of non-physician health workers to manage high mortality diseases like HIV [8]. By promoting skilled, non-physician workers to perform key tasks including case identification, referrals, initiating treatment, and routine physical examinations, the policy makes hypertension management integration into HIV care platforms attainable and sustainable [7]. 

The Nigerian Hypertension Treatment Protocol is a 4-step simplified process for identifying and initiating patients on antihypertension medication treatment, and was developed for use within the primary healthcare system in Nigeria [9] The protocol, when implemented by community health nurses for PLWH in PHCs, allows for the identification and management of hypertension among the population.

In February 2023, the Akwa Ibom State Health Insurance Agency was launched to achieve universal health coverage in the state by providing financial access to qualitative, affordable, equitable and sustainable health care services [5]. Such ongoing efforts to achieve universal health coverage is important to advance the integration of hypertension treatment into HIV care because it facilitates collaboration between healthcare providers in the state as well as cost reduction associated with managing both conditions [10]. 

## Identification of current gaps in hypertension and HIV integrated care in Nigeria

Current gaps in hypertension and HIV care in Nigeria were identified based on specific feedback from the three discussion sessions with the stakeholders. Questions were asked by the MAP-IT research team using a semi-structured focus group format, with the objective of each session being the identification of current gaps in access to hypertension and HIV integrated care and corresponding solutions, from their perspectives. Focus group questions were guided by Community-Based Participatory Research (CBPR) principles [11]. 

The specific gaps identified by the stakeholders were: (1) lack of access to sustained hypertension care for PLWH in Nigeria, despite the burden of HIV-hypertension comorbidity in the country; (2) lack of primary care-level physicians to provide HIV and non-communicable disease integrated care; (3) lack of cost-efficient systems for integrated HIV and non-communicable disease care. Two of the three problems identified by the stakeholders—*the lack of access to sustained hypertension care for PLWH in Nigeria*, and *the lack of primary care-level physicians to provide HIV and non-communicable disease integrated care*—are consistent with the findings of previous studies reporting on barriers to integrated care in the country [12, 13]. The third problem—*lack of cost-efficient systems for integrated HIV and non-communicable disease integrated care*—specifically echoed the recent calls for creating cost-effective healthcare systems in rural and semi-urban areas of Nigeria by Abubakar and colleagues [14].

Following the three stakeholder discussions, five policy recommendations were identified and are documented in this policy brief. Additional detail on these policy recommendations are described in the following section.

## Policy implications

### Mentoring through experience: engaging retired community health nurses

Retired community health nurses represent a wealth of untapped knowledge. By mentoring current community health nurses, retirees can significantly improve the integrated care for HIV and hypertension. This approach not only uses existing resources efficiently but also honors the retirees’ service by involving them in ongoing health system advancements.

### Building bridges: actively linking communities with integrated programs

It is essential to mobilize all community-based organizations and groups to raise awareness on hypertension’s impact on PLWH and to facilitate their access to the nearest clinic providing integrated care. Key players in this initiative include but are not limited to: the Network of People Living with HIV/AIDS in Nigeria, the Association of Women Living with HIV/AIDS in Nigeria, faith-based organizations and leaders, community-based organizations, community pharmacies, schools, traditional birth attendants, and health promotion officers.

### Enhancing skills: integrating hypertension management with HIV education

Incorporating the Nigerian Hypertension Treatment Protocol into the training curriculum of the community health extension workers as a capacity building hypertension management program will bolster their capacity to manage hypertension. This integration ensures that non-physician healthcare providers are well trained to deliver quality care in primary healthcare centers specializing in both HIV and hypertension care for PLWH.

### Widening coverage: improving health insurance accessibility

By embedding educational components into the national insurance schemes, we can expand their reach. Organizing education sessions in community settings, such as religious gatherings or town meetings, will encourage enrollment in these schemes and offer affordable healthcare options. Promoting these schemes alongside existing HIV advocacy efforts can further enhance their visibility and uptake.

### Policy integration: hypertension management within Akwa Ibom State’s primary healthcare system

Capitalizing on the preliminary successes of the MAP-IT study in training community health nurses in hypertension management [9], policymakers in Akwa Ibom State should integrate these practices into the State’s primary healthcare system. This strategy will solidify the foundation for comprehensive hypertension care for PLWH.

## Data Availability

Data sharing is not applicable to this article as no datasets were generated or analyzed during the current study.

## Abbreviations

HIV: Human immunodeficiency virus
MAP-IT: Managing hypertension among people living with HIV: an integrated model
NCD(s): Non-communicable disease(s)
PHC: Primary Health Center
PLWH: People living with HIV
